# Tuberculous Thoracic Aortic Pseudoaneurysm Associated With Spinal Tuberculosis: A Case Report and Literature Review

**DOI:** 10.3389/fmed.2022.882697

**Published:** 2022-05-26

**Authors:** Yurou Chen, Bo Sheng, Jia Li, Furong Lv

**Affiliations:** Department of Radiology, The First Affiliated Hospital of Chongqing Medical University, Chongqing, China

**Keywords:** spinal tuberculosis, thoracic aortic pseudoaneurysm, endovascular repair, endovascular stent-graft, case report

## Abstract

**Background:**

Thoracic aortic pseudoaneurysm associated with spinal tuberculosis is a rare but fatal condition. The risk of pseudoaneurysm rupture is extremely high and this disease needs greater awareness. The present study reported a case of thoracic aortic pseudoaneurysm caused by paravertebral cold abscess with spinal tuberculosis.

**Case presentation:**

A 35-year-old woman with back pain was diagnosed with thoracic aortic pseudoaneurysm with spinal tuberculosis, and endovascular aneurysm repair (EVAR) was performed. The patient's symptoms disappeared after EVAR, following which she was discharged.

**Conclusions:**

The case highlighted that in cases where non-enhanced computed tomography (CT) revealed that the aortic vessel was surrounded by a paravertebral abscess, magnetic resonance imaging (MRI) should be performed to confirm whether the presence of a pseudoaneurysm. Upon diagnosis of pseudoaneurysm, surgery should be performed immediately. In recent times, EVAR has emerged as a promising alternative to open surgery.

## Introduction

Spinal tuberculosis is the most common form of osteoarticular tuberculosis and there had been a significant increase in the incidence of spinal tuberculosis in developing countries over the past few years. However, incidences of thoracic aortic pseudoaneurysms associated with spinal tuberculosis are rare (1, 2). It has been previously reported that aortic pseudoaneurysm exposes patients to a very high risk of unpredictable rupture (3).

The present study reported a case of thoracic aortic pseudoaneurysm caused by paravertebral cold abscess with spinal tuberculosis and discussed the clinicoradiological features and treatment of the disease.

## Case Description

**Figure 1:**
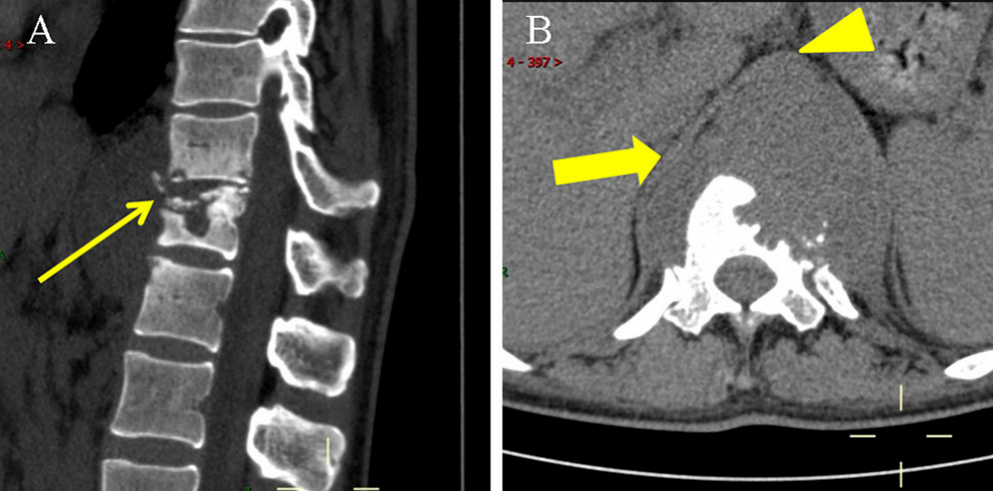
Non-enhanced CT scan on 13 October 2021. **(A)** Lateral CT scan showed T11-L1 with bone destruction and sequestrums (thin arrow). **(B)** Axial CT scan showed paravertebral abscess (thick arrow) was indistinguishable from the thoracic aorta (arrowhead).

A 35-year-old woman was admitted in October 2021 with a history of low back pain for the past year, which worsened over the past 2 weeks, and the patient complained of radiating pain in the left lower limb. The patient was treated at another hospital 2 weeks ago, where MRI, CT, and x-ray showed T11–L1 bone destruction. She was diagnosed with thoracic spine tuberculosis and the details of the diagnosis were not mentioned. Then she was treated with isoniazid, rifampin, and ethambutol. The laboratory examination revealed white blood cell count (WBC) 2.10× 10^9^ /L, erythrocyte sedimentation rate (ESR) 38 mm/h, C-reactive protein (CRP) 10.7 mg/L, interleukin-6 (IL-6) 0.35 pg/ml, and D-dimer 1.88 mg/L FEU. The patient tested positive for tuberculosis infection, as assessed by a T cell spot test. Chest CT showed scattered grain-like nodules in both lungs, suggesting pulmonary tuberculosis. CT of thoracic and lumbar spine revealed bone destruction of T11–L1, which was obvious in T12, mild reduction in the height of the vertebral body and narrowing of T11-12 intervertebral space, with a cold paravertebral abscess on both sides (Figure 1). These results were consistent with spinal tuberculosis. Consequently, the initial diagnosis was spinal tuberculosis with a cold abscess (T11–L1). Further, an ultrasound revealed an abnormal echo in the left paracolonic groove, which was associated with a cold abscess. Consequently, the patient was subjected to ultrasound-guided percutaneous catheter drainage on 15th October 2021 and 600 ml of pus was drained.

**Figure 2:**
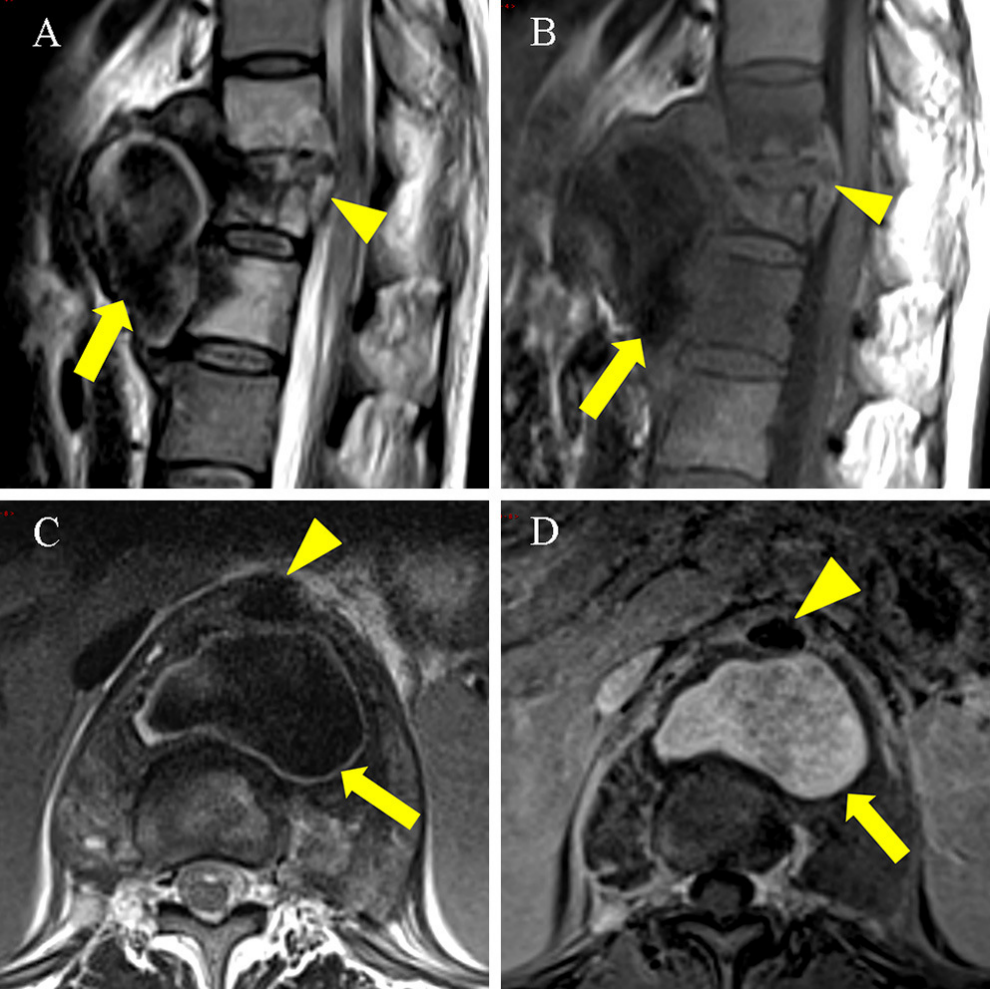
An enhanced MRI scan on 6 November 2021 showed a pseudoaneurysm measuring 5.7 × 3.7 × 5.8 cm. **(A)** (T2WI), **(B)** (T1WI): Sagittal MRI scan showed T11-L1 with bone destruction (arrowhead). Large cold abscess in the front of the vertebral body with flow void signal (thick arrow). **(C)** T2WI axial MRI scan showed a cold abscess with flow void signal (thick arrow) located posterior to the thoracic aorta (arrowhead). **(D)** Axial enhanced MRI scan showed the pseudoaneurysm (thick arrow) located posterior to the thoracic aorta (arrowhead) was hyperintense.

After 16 days of drainage, the patient complained of severe chest and back pain. An electrocardiogram (ECG) was performed and it showed no any abnormal finding. The laboratory examination revealed WBC 3.40 × 10^9^ /L, ESR 47 mm/h, CRP 17.4 mg/L, IL-6 37.5 pg/ml, PCT 0.07 ng/ml, and D-dimer 1.89 mg/L FEU. Re-examination of the thoracic and lumbar spine using enhanced MRI revealed bone destruction in T11–L1 and a massive mixed density shadow was observed in the front, wherein blood flow showed low signal on T1WI and T2WI, primarily owing to flow void effect. Obvious enhancement was observed after the lesion was enhanced (Figure 2), which was indicative of a thoracic aortic pseudoaneurysm. Consequently, it was recommended that endovascular aneurysm repair must be performed immediately, however, only bone graft fusion and internal fixation were performed, owing to the patient's personal reasons, and vertebral biopsy revealed a negative result in the acid-fast staining and the culture was positive for *M. tuberculosis*.

**Figure 3:**
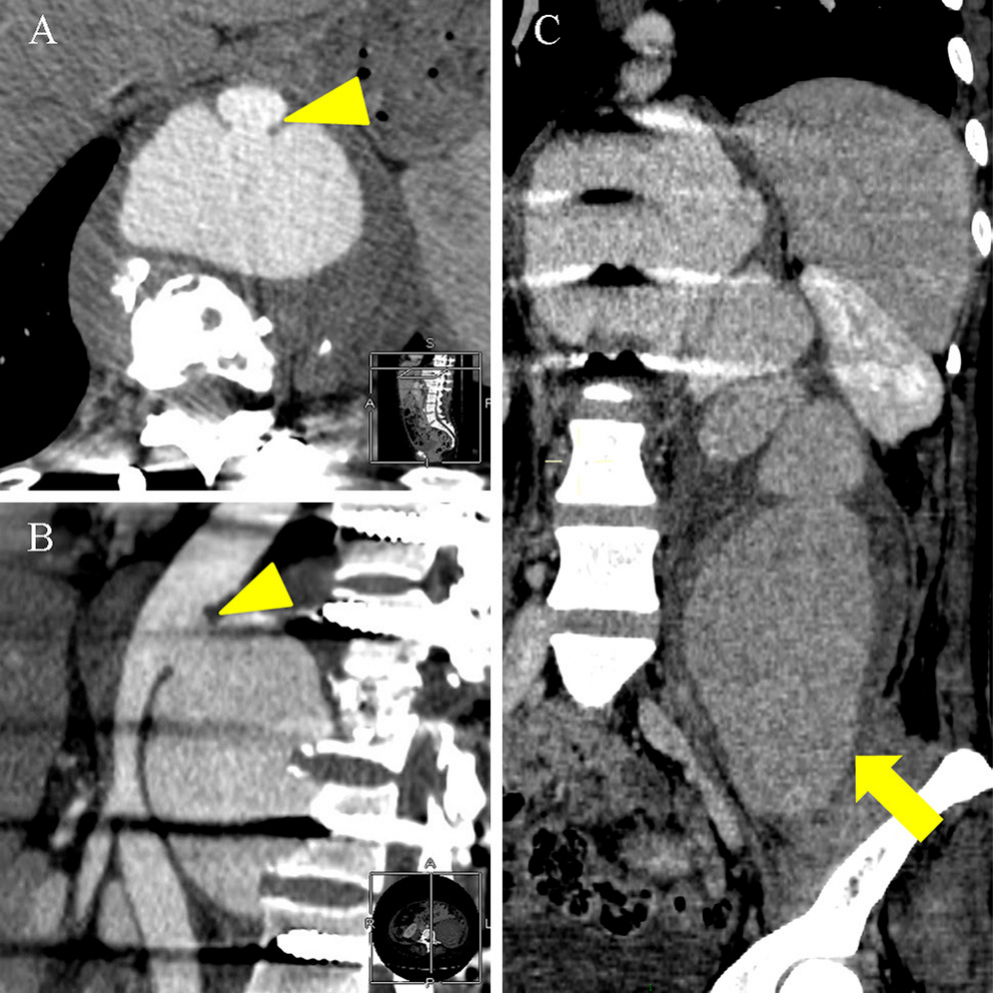
An enhanced CT scan on 1 December 2021 showed a pseudoaneurysm measuring 8.2 × 5.2 × 22.9 cm. **(A,B)** Axial and lateral enhanced CT scan showed the thoracic aorta communicated with the pseudoaneurysm through the rupture (arrowhead). **(C)** Coronal enhanced CT scan showed the pseudoaneurysm significantly enlarged into the left iliac fossa (thick arrow) compared with Enhanced MRI scan on 6 November 2021.

**Figure 4:**
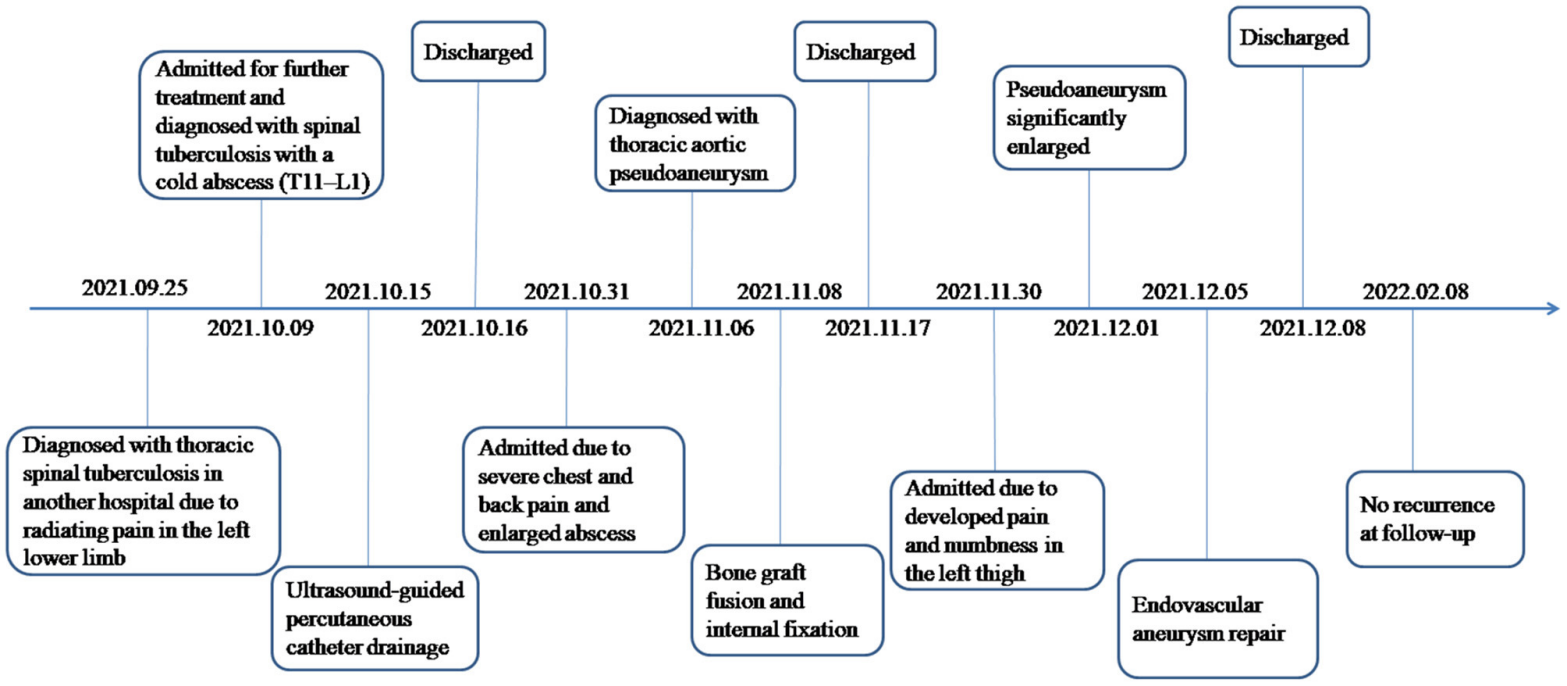
A timeline showed the progress of the disease and the patient's treatment and follow-up.

Further, the patient developed pain and numbness in the left thigh after 10 days of the procedure. The laboratory examination revealed WBC 4.80 × 10^9^ /L, ESR 26 mm/h, CRP 14.4 mg/L, IL-6 9.49 pg/ml, and D-dimer 10.42 mg/L FEU.A CT scan revealed enlargement of pseudoaneurysm (Figure 3). Following this, the patient underwent endovascular aneurysm repair. Digital subtraction angiography was performed with the patient under general anesthesia, which revealed an approximate 10 mm breach on the lower portion of the descending aorta, and the orifice of the pseudoaneurysm to be 20 mm away from the lower part of the celiac trunk. The stent graft (Medtronic Inc., 25 mm in diameter and 70 mm in length) was positioned well and correctly and repeated angiography confirmed a complete closure of the mass, a smooth blood flow in the aorta, and a firmly fixed stent on the aortic wall without distortion and displacement; no peripheral leakage of the contrast agent was observed. The surgery was successful and the patient continued to receive isoniazid (0.3 g/day), rifampin (0.45 g/day), and ethambutol (0.75 g/day) during the follow-up. The patient's symptoms gradually disappeared, and she was discharged. After 3 months' follow-up, repeated CT showed a firmly fixed stent on the aortic wall without distortion and displacement and a smooth blood flow in the aorta, and the pseudoaneurysm was basically absorbed without recurrence. Because the follow-up was outpatient, no laboratory examinations were done (Figure 4).

## Discussion

Tuberculous pseudoaneurysm is a rare disease, and the case with spinal tuberculosis is even rarer. We reviewed previous literature from 1999 to the present and found 21 patients in 17 related literature with tuberculous pseudoaneurysm combined with spinal tuberculosis (2–18) (Table 1).

**Table 1:** Review of literature about tuberculous pseudoaneurysm associated with vertebral tuberculosis.

**Patient no**	**References**	**Literature type**	**Location** **(artery)**	**Location** **(vertebrae)**	**Other infected organs**	**Sugical procedure**	**Anti-TB**	**Follow-up** **month**	**Outcome**
1	Long et al. (4)	Review	Infrarenal abdominal aorta	/	Lung	Axillobifemoral graft	Yes	/	/
			Suprarenal abdominal aorta	T11-12	Lung	Dacron aortic interposition graft	Yes	/	/
2	Liu et al. (5)	Case report	Suprarenal abdominal aorta	/	/	Endovascular sten graft	Yes	24	No recurrence
3	Choudhary et al. (6)	Case report	Descending thoracic aorta	/ L4-5	Lung	Vascutek graft	Yes	20	No recurrence
			Infrarenal aorta		Lung	Vascutek tube graft	Yes	24	No recurrence
4	Pimple et al. (7)	Case report	Descending thoracic aorta	T5-8	/	Refused surgery	/	Lost	/
5	Dahl et al. (8)	Case report	Abdominal aorta	L3-4	/	Bifurcated vascular graft	Yes	9	No recurrence
6	Falkensammer et al. (9)	Case report	Thoracoabdominal aorta	T12-L1	Lung	Aortic homograft	Yes	15	No recurrence
7	Jain et al. (10)	Case report	Thoracoabdominal aorta	T7-L2	/	Endoaneurysmorrhaphy	Yes	33	No recurrence
8	Han et al. (11)	Case report	Abdominal aorta	L2	/	EVAR(endovascular aneurysm repair)	Yes	/	No recurrence
9	Takahashi et al. (12)	Case report	Descending thoracic aorta	T8-10	/	*In situ* reconstruction with a prosthetic graft	Yes	24	No recurrence
10	Husein et al. (13)	Case report	Infrarenal abdominal aorta	L3-4	/	*In situ* silver-coated bifurcated Dacon graft	Yes	12	No recurrence
11	Li et al. (14)	Review	Descending thoracic aorta	T3-5	Lung	EVAR	Yes	12	No recurrence
12	Villegas et al. (15)	Case report	Abdominal aort	L2-4	/	EVAR	Yes	36	No recurrence
13	Liu et al. (16)	Case report	Infrarenal abdominal aorta	L4	/	*In situ* placement of a prosthetic graft	Yes	6	No recurrence
14	Zhang et al. (3)	Review	Infrarenal abdominal aorta	L3-5	/	EVAR	Yes	6	No recurrence
15	Xue et al. (17)	Case report	Descending thoracic aorta	T4-5	Lung	Refused surgery	Yes	Death	Death
			Thoracic aorta	T5-11	Lung	Thoracic surgery	/	Death	Death
			Thoracic aorta	T6-10	Lung	Endovascular stent graft	Yes	18	No recurrence
16	Tang et al. (2)	Review	Thoracic aorta	T3-5	Lung	Endovascular stent graft	Yes	32	No recurrence
17	Li et al. (18)	Review	Descending thoracic aorta	T11-12	Lung	EVAR	Yes	24	No recurrence

It has been previously reported that the formation mechanisms involved in tuberculous pseudoaneurysm might include bacterial spread through the lymphatic vessels around the arteries, direct inoculation of *M. tuberculosis* after vasculature trauma, *M. tuberculosis* invasion to the wall of the blood vessel through feeding vessel, and direct invasion and spread of the bacteria from lymph nodes, abscess, and bone tuberculosis around the artery (19). The dominant mechanism is erosion through the aorta of a contiguous focus of disease, accounting for 75% of cases (4). Tuberculous lymphadenitis, pericarditis, empyema, spondylitis, or paravertebral abscess destroy the entire thickness of the aortic wall, then hemorrhage and perivascular hematoma formation occur, the hematoma is enwrapped and retain communication with the lumen, the pseudoaneurysm is formed (20). Perhaps this is why most tuberculous aneurysms are pseudotuberculous, accounting for 86% (6). In the present study, according to the clinical manifestations during the onset of the patient, and the CT and MRI scans revealed the presence of tuberculous damage around the pseudoaneurysm, which was accompanied by an abscess. Thus, it was believed that a pseudoaneurysm might be formed by an abscess invading the aorta.

In general, pseudoaneurysms can occur in any part of the arterial system (10), particularly in the ascending aorta, distal aortic arch, proximal descending thoracic aorta, distal descending thoracic aorta, and infrarenal abdominal aorta (6). In a previous study conducted by Long et al. (4), 19 pseudoaneurysms were found to be located in the thoracic aorta, 21 in the abdominal aorta, and only one was located at the junction of the thoracic aorta and abdominal aorta. Following this, Forbes et al. added 15 more patients. For these patients, nine pseudoaneurysms were located in the thoracic aorta, five in the abdominal aorta, while one was located at the junction of the thoracic and abdominal aorta (21). In the present case, it was located in the thoracic aorta. The occurrence of fewer cases at the junction of the thoracic and abdominal aorta might be attributed to a lower incidence of atherosclerosis. In addition to this, fewer lymph nodes are located around this section of the aorta, and also the aorta is separated from its neighboring structures through the diaphragm (4).

The clinical presentations of most of the patients usually involve the constitutional symptoms and signs of tuberculosis with or without an aneurysmal mass effect, depending on the location. Tuberculous aortic aneurysm usually manifests as a pulsatile or palpable mass, chest pain, dysphagia, hoarsenees, abdominal pain, back pain, and if complicated, by a fistula, perforations, bleeding, and rupture (22). In the present study, the patient's third admission was due to the symptoms of aortic pseudoaneurysm. In particular, the patient suffered from back pain and progressive decrease in hemoglobin, and the presence of a huge mass in the left iliac fossa was reported, which was consistent with previous studies. However, except for a small number of giant aneurysms with pulsating masses on the body surface, other pseudoaneurysms lack clinical special symptoms and signs and are easily misdiagnosed in clinical practice. Thus, a pseudoaneurysm should always be considered if a diagnosed case of tuberculosis deteriorates suddenly or develops pressure symptoms due to presence of a mass (6).

In the past few years, advances in imaging technology have improved the diagnosis of aortic pseudoaneurysm. In particular, a CT scan can be used to detect spinal cord compression, paravertebral abscess, and degree of vertebral body damage. Moreover, the detection rate of pseudoaneurysm by enhanced CT is very high. In such cases, MRI might provide better visualization of surrounding soft tissue injuries, spinal cord compression, and pseudoaneurysms (2). The typical MRI features of aortic pseudoaneurysm include aortic communication with the mass through rupture, elicitation of mixed signals from the mass, and presence of flow void effect in the mass. In the present study, only a non-enhanced CT was performed before drainage, which showed the presence of a paravertebral abscess. Since no enhanced CT or enhanced MRI was done, pseudoaneurysm was missed. Therefore, in cases where the aorta is surrounded by a paravertebral abscess on CT and a flow void signal is visible in the cold abscess on MRI, enhanced CT and enhanced MRI should be performed to confirm the occurrence of pseudoaneurysm.

Once pseudoaneurysm is diagnosed, surgery is a must, regardless of its size, because even pseudoaneurysm with a diameter of 1 cm can cause rupture (4). Currently, surgery combined with simultaneous anti-tuberculosis drug treatment should be used for the disease (3, 21), and no evidence showing that only anti-tuberculosis drug treatment or surgery alone can achieve a cure exists (4, 6). The surgical repair of tuberculous pseudoaneurysm was first tried by Herdon et al. (23), and the patient died on the 6th-day post-surgery. In 1959, Rob and Eastcott (24) reported the first successful reconstruction of tuberculous aortic aneurysm, which used Orlon cloth graft. The currently available treatment methods include open intervention, which involves direct suture closure, patch repair, vascular lesion removal, and synthetic vascular replacement, and extra-anatomic reconstruction, or minimally invasive surgeries that involve embolization and endovascular aneurysm repair (EVAR) (2). Open surgery has always been the gold standard for the treatment of tuberculous pseudoaneurysm, but recently EVAR has been widely used as an alternative to open surgery (14, 15). In particular, the use of EVAR avoids a large incision, cardiopulmonary bypass, aortic cross-clamping, negative effects on respiratory function, and transfusions (25). However, EVAR is also associated with certain disadvantages, such as infection at the transplantation site, late prosthesis rupture, and embolism (5). Dogan et al. reported successful rescue of a patient with a ruptured tuberculous hemangioma using an endovascular stent graft (26). However, in another study, Xue et al. reported rupturing of pseudoaneurysm during the operation, and the patient died (17). The incidence of tuberculosis/aneurysm recurrence is quite close between the open surgery and EVAR, which may indicate that surgical choice does not have an effect on the postoperative recurrence of tuberculosis/aneurysm (27), thus the choice of surgical approaches should depend on the patients' specific conditions. In patients with clinical acute hemorrhage, age over 60 years, immunosuppressive status, malnutrition, and weight loss, EVAR is an attractive option. While the main problem with EVAR is that debridement of the infected tissue cannot be done, which are main parts of the surgical strategy. This debridement likely plays a key role in eliminating necrotic tissue and, thus, improving antimycobacterial drug efficiency (28). Therefore, if the patient's condition permits, open surgery should be performed to ensure infected sources including aortic wall, and abscess, are able to be completely debrided.

In the study of Xue et al., two cases with tuberculous pseudoaneurysms combined with spinal tuberculosis died. One patient refused surgery and received only anti-tuberculosis treatment. Unfortunately, the patient died of sudden massive hemoptysis 2 years after discharge; the other patient underwent spinal surgery first, but the pseudoaneurysm ruptured suddenly during the procedure and the patient died (17). The case reported in the present study was in poor condition at the second hospitalization. It was recommended to treat the aortic pseudoaneurysm first to reduce the possibility of rupture and then perform vertebral surgery. However, only bone graft fusion and internal fixation were performed, owing to the patient's reasons. Consequently, pseudoaneurysm enlarged rapidly due to decreased pressure after drainage, the enlarged pseudoaneurysm then compressed the nerves, and causing pain in the left thigh. The patient underwent EVAR immediately, and the pseudoaneurysm was basically absorbed without recurrence after 3 months' follow-up. With these cases, we deeply realize that pseudoaneurysm is at risk of unpredictable rupture no matter how small it is, thus it is important to repair the pseudoaneurysm as soon as possible. Following this, further surgery can be conducted to treat the spine.

Our case has some characteristics: In our case, the pseudoaneurysm communicated with the cold abscess, and due to the pressure of the cold abscess, the pseudoaneurysm did not enlarge significantly at the beginning. Only a small flow void signal measuring 2.0 × 1.3 cm can be found on MRI at first admission (before drainage), while the radiologist focused more on the spinal tuberculosis and the large cold abscess, and the mixed signal of the cold abscess itself interfered with the identification of the flow void signal, so the flow void signal was not considered and no further diagnostic examination was done to confirm the diagnosis of pseudoaneurysm. The surgeon performed cold abscess drainage on the patient, after which the cold abscess was drained, the pseudoaneurysm enlarged rapidly due to the sudden decrease in pressure, with the patient's back pain and chest pain worsened, and the pseudoaneurysm was diagnosed at the second admission. In fact, abscess drainage at first admission was extremely risky, even the patient was once at risk of death. After summing up the experience of the complete diagnosis and treatment process of this patient, as well as literature review, we believe that although the incidence of spinal tuberculosis associated with pseudoaneurysms is rare, patients with spinal tuberculosis must be carefully observed for adjacent aorta, especially if there is a cold abscess encasing the aorta, be sure to observe for a flow void effect in and around the cold abscess. If a flow void signal is found, regardless of its size, enhanced MRI should be performed to confirm, so that the pseudoaneurysm can be repaired early and actively.

In addition, to our best knowledge, most of the currently available literature on tuberculous pseudoaneurysm is limited to case reports, and tuberculous pseudoaneurysm is so rare that can be easily missed in clinical practice. The main purpose of reporting this case was to draw great attention to tuberculous pseudoaneurysm so that early diagnosis and active treatment of the disease can be achieved.

## Conclusion

In conclusion, the occurrence of tuberculous pseudoaneurysm is very rare, but we should pay great attention to the disease in clinical practice, and the possibility of pseudoaneurysm should be considered especially for patients with spinal tuberculosis combined with paravertebral abscesses and persistent chest and back pain. Once pseudoaneurysm is suspected, examinations should be performed immediately to confirm the diagnosis. In cases where the aorta is surrounded by a paravertebral abscess and a flow void signal is visible in the cold abscess on MRI, enhanced CT and enhanced MRI should be performed to confirm the occurrence of pseudoaneurysm. When pseudoaneurysm is diagnosed, surgery should be performed immediately combined with anti-tuberculosis drugs treatment, regardless of its size, otherwise the patient will be put at risk of unpredictable and fatal rupture. Moreover, pseudoaneurysm might be treated before surgery for vertebral tuberculosis to prevent fatal rupture.

## Data Availability Statement

The original contributions presented in the study are included in the article/supplementary material, further inquiries can be directed to the corresponding author/s.

## Ethics Statement

Written informed consent was obtained from the individual(s) for the publication of any potentially identifiable images or data included in this article.

## Author Contributions

YC collected the patient's information and wrote the manuscript. BS and FL analyzed patient data. JL revised the manuscript. All authors have read and approved the final manuscript.

## Conflict of Interest

The authors declare that the research was conducted in the absence of any commercial or financial relationships that could be construed as a potential conflict of interest.

## Publisher's Note

All claims expressed in this article are solely those of the authors and do not necessarily represent those of their affiliated organizations, or those of the publisher, the editors and the reviewers. Any product that may be evaluated in this article, or claim that may be made by its manufacturer, is not guaranteed or endorsed by the publisher.
